# FISH Mapping of Telomeric and Non-Telomeric (AG_3_T_3_)_3_ Reveal the Chromosome Numbers and Chromosome Rearrangements of 41 Woody Plants

**DOI:** 10.3390/genes13071239

**Published:** 2022-07-14

**Authors:** Xiaomei Luo, Zhoujian He, Juncheng Liu, Hongyi Wu, Xiao Gong

**Affiliations:** College of Forestry, Sichuan Agricultural University, Huimin Road 211, Wenjiang District, Chengdu 611130, China; hezhouj@163.com (Z.H.); juhnson@foxmail.com (J.L.); qywuhy@163.com (H.W.); gongxiao202209@163.com (X.G.)

**Keywords:** TTTAGGG, karyotype asymmetry, telomere, chromosome reorganization

## Abstract

Data for the chromosomal FISH mapping localization of (AG_3_T_3_)_3_ are compiled for 37 species belonging 27 families; for 24 species and 14 families, this is the first such report. The chromosome number and length ranged from 14–136 and 0.56–14.48 μm, respectively. A total of 23 woody plants presented chromosome length less than 3 μm, thus belonging to the small chromosome group. Telomeric signals were observed at each chromosome terminus in 38 plants (90.5%) and were absent at several chromosome termini in only four woody plants (9.5%). Non-telomeric signals were observed in the chromosomes of 23 plants (54.8%); in particular, abundant non-telomeric (AG_3_T_3_)_3_ was obviously observed in *Chimonanthus campanulatus*. Telomeric signals outside of the chromosome were observed in 11 woody plants (26.2%). Overall, ten (AG_3_T_3_)_3_ signal pattern types were determined, indicating the complex genome architecture of the 37 considered species. The variation in signal pattern was likely due to chromosome deletion, duplication, inversion, and translocation. In addition, large primary constriction was observed in some species, probably due to or leading to chromosome breakage and the formation of new chromosomes. The presented results will guide further research focused on determining the chromosome number and disclosing chromosome rearrangements of woody plants.

## 1. Introduction

Most eukaryotes possess chromosomal termini made up of 5–8 bp simple tandem DNA repeats—commonly called telomeric repeats [1,2,3]—which, in all eukaryotes, share similarities. Telomeric DNA repeats typically follow the formula (T_x_A_y_G_z_)_n_. Furthermore, they are well-conserved, and previous findings have revealed their diversity. There exist at least 17 variable telomeric sequences: TAG_3_ [4], TA_2_G_2_ [5], TA_2_G_3_ [5], T_2_AG_2_ [6], T_2_AG_3_ [7], T_3_AG_3_ [8,9,10], AG_3_T_3_ [11], T_4_AG_3_ [12], A_2_TG_6_ [13], T_2_AT_2_AG_3_ [7], T_6_AG_3_ [14,15], TCAG_2_ [16], T_2_AG_2_C [17], T_2_CAG_2_ [18], T_3_CAG_2_ [18], C_3_TA_3_ [19], T_2_N_4_AG_3_ [20], and T_2_ATG_3_CTCG_2_ [21]. Undoubtedly, the most widespread telomeric repeat is T_2_AG_3_ in animals [22], while that in most plants is T_3_AG_3_ [23].

Telomeric repeats are not often localized at chromosomal termini and have also been found in multiple intercalary sites of chromosomes in many species [3,23,24,25]. Interstitial telomeric sequences may represent a significant part of the telomeric DNA [3]. Cytogenetic analysis has found two major types of interstitial telomeric sequences: one is heterochromatic and large, found in centromeric or pericentromeric regions, while the other type is short and distributed at various positions in chromosomes [3]. Unlike telomeric repeats located at termini, interstitial telomeric sequences confer karyotype plasticity. Their unstable and high-length polymorphisms may increase chromosomal fragility and contribute to chromosomal reorganization [3,23,24]. Interstitial telomeric sequences have been shown to cause a change in the chromosome number in *Cardamine cordifolia* A. Gray [26], as well as in Asteraceae and Brassicaceae [27]. Interstitial telomeric sequences may be derived from centric fusions of Robertsonian translocation [28,29,30], generated through pericentric inversions [31,32], or be the product of equilocal dispersion of telomeric DNA to interstitial regions of the chromosome through transposition or heterologous recombination [33,34].

Many studies have reported on the T_3_AG_3_ distribution in woody plants. T_3_AG_3_ were observed at each chromosome end in *Populus trichocarpa* Torr. and A. Gray ex Hook. [35], *Picea abies* (L.) H. Karst. and *Larix decidua* Mill. [36], *Abies alba* Mill. [37,38], *Cycas revoluta* Thunb. [39], *Strobus* spp. [40], *Aralia elata* (Miq.) Seem., *Dendropanax morbiferus* H. Lév., *Eleutherococcus sessiliflorus* (Rupr. Et Maxim.) Seem. and *Kalopanax septemlobus* (Thunb. ex A.Murr.) Koidz [41]. Furthermore, T_3_AG_3_ has been observed at the interstitial regions and at all chromosome ends in *Pinus* spp. [42,43,44,45] and *Podocarpus* spp. [46]. No T_3_AG_3_ signal was found in species of *Sessea*, *Vestia*, and *Cestrum*, but the T_3_AG_3_ sequence was dispersed in the *Cestrum* genome [14,15].

AG_3_T_3_ is similar to but different from T_3_AG_3_ and has only been found to be used in 22 woody plants, 14 species, and 11 genera. AG_3_T_3_ have been observed at each chromosome end in *Hibiscus mutabilis* L. [47]; *Juglans regia* L. ‘Chuanzao1’, *J. regia* L. ‘Yanyuanzao’, *Juglans sigillata* Dode ‘Maerkang’, and *J. sigillata* Dode ‘Muzhilinhe’ [48]; *Fraxinus pennsylvanica* Marsh., *Syringa oblata* Ait., *Ligustrum lucidum* Lindl. and *Ligustrum* × *vicaryi* Rehder [49]; and *Berberis diaphana* Maxim. and *Berberis soulieana* Schneid [50]. Telomeric AG_3_T_3_ ensures integrated and easily countable chromosomes. Furthermore, T_3_AG_3_ has been observed in the interstitial regions and at all chromosome ends in *Hippophaë rhamnoides* ‘Wucifeng’, *H. rhamnoides* ‘Shenqiuhong’, *H. rhamnoides* ‘Zhuangyuanhuang’, cultural *H. rhamnoides* ssp. *sinensis*, wild *H. rhamnoides* ssp. *sinensis* [11], *Robinia pseudoacacia* L., *R. pseudoacacia* ‘idaho’, *R. pseudoacacia* f. *decaisneana* (Carr.) Voss, *Styphnolobium japonicum* (L.) Schott, *Amorpha fruticose* L. [51], and *C. campanulatus* R.H. Chang and C.S. Ding [52]. Non-telomeric AG_3_T_3_ may indicate chromosomal variation. There is still a great need to explore the telomeric and non-telomeric AG_3_T_3_ chromosome distribution in other woody plants. To determine the chromosome number and disclose the chromosome rearrangements in woody plants, in this study, we carried out fluorescent in situ hybridization (FISH) mapping to reveal highly abundant (AG_3_T_3_)_3_, established an ideogram to describe the complex genome architecture and, finally, discussed the proposed origin of (AG_3_T_3_)_3_ diversity in woody plants.

## 2. Materials and Methods

The species chosen for these experiments were firstly considered due to the occurrence of karyotype rearrangements [11,47,48,49,50,51,52,53], and secondly for investigation of species in which (AG_3_T_3_)_3_ has not yet been explored. *Zea mays* L. was chosen as it possesses the telomeric repetitive unit AGGGTTT conserved in plant chromosome telomeres. It is contained in the sequence named M8-2D, a B chromosome-specific sequence in *Z. mays*, which has low homology to clones from *Z. mays* chromosome 4 centromere. M8-2D is localized in B chromosome centromeric and telomeric regions [53]. Hence, we used *Z. mays* to test the used probe and as a control.

Details of the seeds or seedlings of *Z. mays* and the 41 woody plants (belonging to 37 species, 27 genera, 18 families) used in the present work are provided in Table 1. All 42 plants were collected from 12 Counties or Districts of Sichuan Province, China. 

### 2.1. Probe and Chromosome Preparation

The 21 bp oligo-probe of (AG_3_T_3_)_3_, 5′-AGGGTTTAGGGTTTAGGGTTT-3′, was first reported in *Z. mays* [53] and has been further applied in Berberidaceae [50], Calycanthaceae [52], Elaeagnaceae [11], Fabaceae [51], Juglandaceae [48], Malvaceae [47], and Oleaceae [49]. This oligo-probe was synthesized by Sangon Biotech Co., Ltd. (Shanghai, China), and tested simultaneously in a single round of FISH. The oligo-probe was 5′-labeled with 6-carboxy-fluorescein (6-FAM; absorption/emission wavelengths 494 nm/518 nm; green).

Collected seeds were germinated in culture dishes with wet filter paper and kept at 25 °C in the daytime and at 18 °C in the night until the roots were ~2 cm in length, which were then cut. The collected seedlings were cultured in soil at room temperature (15–25 °C) until many new roots grew out, which were then cut again. The cut roots were treated with nitrous oxide (N_2_O) gas for 2–6 h, with treatment time depending on chromosome length and cell wall lignification. Next, the samples were fixed in glacial acetic acid for 5–10 min, then kept in 75% ethyl alcohol. Chromosome preparation was carried out according to the procedure described by Luo et al. [51]. As these techniques have been described elsewhere, they will be detailed briefly here. Approximately 1 mm of the meristematic zone of the root tip was enzymolyzed at 37 °C for 45 min by using cellulase and pectinase (1 mL buffer + 0.04 g cellulase + 0.02 g pectinase, the buffer 50 mL was included 0.5707 g trisodium citrate + 0.4324 g citric acid), which were produced by Yakult Pharmaceutical Ind. Co., Ltd. (Tokyo, Japan) and Kyowa Chemical Products Co., Ltd. (Osaka, Japan), and then mixed into suspension for dropping onto slides. These slides were air dried then examined using an Olympus CX23 microscope (Olympus, Tokyo, Japan). 

### 2.2. FISH Hybridization

Slides with well-spread samples were used for hybridization. Chromosomes were first subjected to a series of fixation (10 min, 4% paraformaldehyde, room temperature), dehydrated (5 min, 75%, 95%, 100% ethanol, room temperature), denatured (2 min, deionized formamide, 80 °C), and a second dehydration (5 min, 75%, 95%, 100% ethanol, −20 °C), and then hybridized (10 μL hybridization mixture of 0.375 μL of (AG_3_T_3_)_3_, 4.675 μL of 2× SSC, and 4.95 μL of ddH_2_O) for 1–2 h at 37 °C. Subsequently, hybridized chromosomes were washed with 2× SSC and ddH_2_O twice for 5 min at room temperature, and air-dried. Finally, they were counterstained with 4,6-diamidino-2-phenylindole (DAPI, Vector Laboratories, Inc., Burlingame, CA, USA) for 5 min, referring to the procedure described by Luo et al. [51]. Slides were checked using an Olympus BX-63 microscope (Olympus Corporation, Tokyo, Japan), and FISH images were captured by a DP-70 CCD camera connected to the microscope. 

### 2.3. Karyotype Analysis

Raw images were processed using the DP Manager (Olympus Corporation, Tokyo, Japan) and Photoshop CC 2015 (Adobe Systems Incorporated, San Jose, CA, USA) software. At least ten slides of each plant were observed, and at least fifteen cells with good chromosome spread were used for chromosome counting and length measurement. All chromosomes were aligned from longest to shortest. The chromosome ratio was determined by comparing the length of the longest chromosome to that of the shortest chromosome. Further karyotype analysis could not be carried out due to the small chromosome size and unclear centromere location of many of the species.

## 3. Results

### 3.1. Karyotype Analysis Revealed Differences among 37 Species

**Figure 1:**
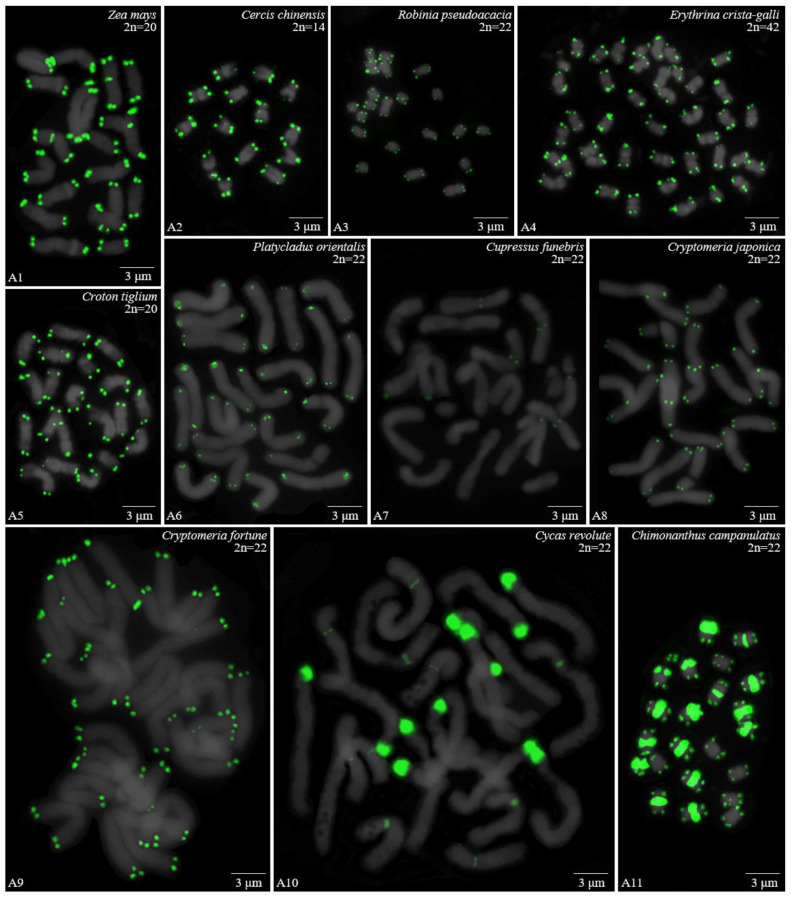
Oligo-FISH depicting the (AG_3_T_3_)_3_ present in *Z. mays* and ten woody plants. Chromosomes in A1–A8 and A11 are from metaphase, while the chromosomes in A9–A10 are from prometaphase. Oligo-probe (AG_3_T_3_)_3_ is exhibited by green signals: (**A1**) *Z. mays*, 2n = 20; (**A2**) *C. chinensis*, 2n = 14; (**A3**) *R. pseudoacacia*, 2n = 22; (**A4**) *E. crista-galli*, 2n = 42; (**A5**) *C. tiglium*, 2n = 20; (**A6**) *P. orientalis*, 2n = 22; (**A7**) *C. funebris*, 2n = 22; (**A8**) *C. japonica*, 2n = 22; (**A9**) *C. fortunei*, 2n = 22; (**A10**) *C. revoluta*, 2n = 22; and (**A11**) *C. campanulatus*, 2n = 22. Bar: 3 μm.

**Figure 2:**
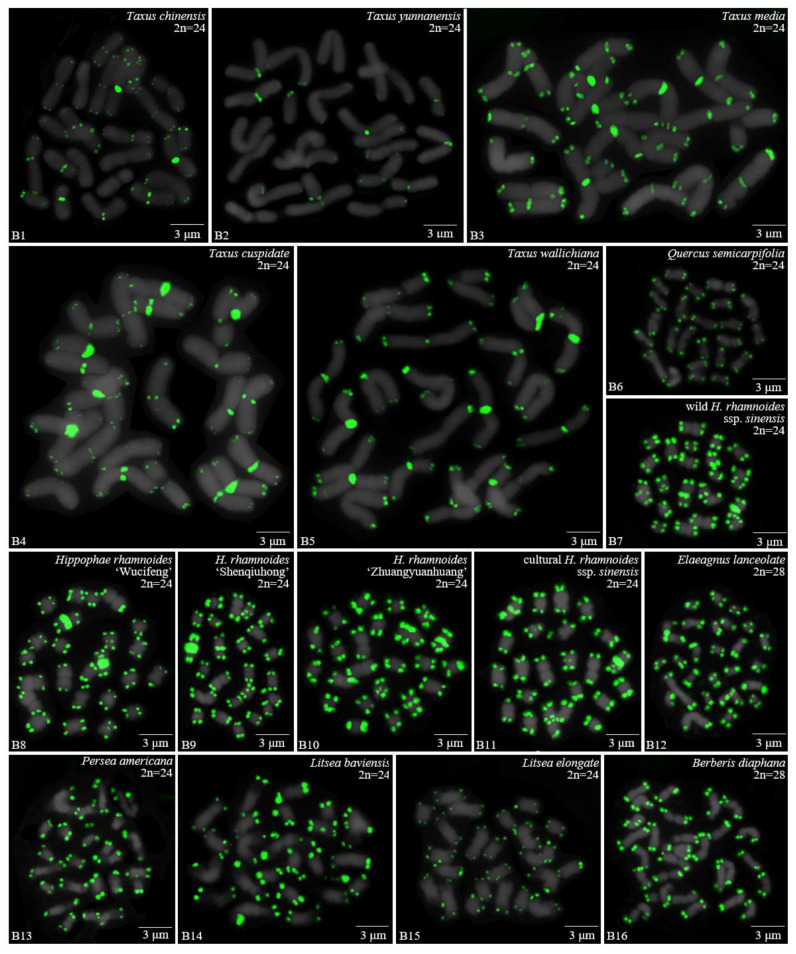
Oligo-FISH depicting the (AG_3_T_3_)_3_ present in 16 woody plants. All chromosomes in B1–B16 are from metaphase. Oligo-probe (AG_3_T_3_)_3_ is exhibited by green signals: (**B1**) *T. chinensis*, 2n = 24; (**B2**) *T. yunnanensis*, 2n = 24; (**B3**) *T. media*, 2n = 24; (**B4**) *T. cuspidata*, 2n = 24; (**B5**) *T. wallichiana*, 2n = 24; (**B6**) *Q. semecarpifolia*, 2n = 24; (**B7**) wild *H. rhamnoides* ssp. *sinensis*, 2n = 24; (**B8**) *H. rhamnoides* ‘Wucifeng’, 2n = 24; (**B9**) *H. rhamnoides* ‘Shenqiuhong’, 2n = 24; (**B10**) *H. rhamnoides* ‘Zhuangyuanhuang’, 2n = 24; (**B11**) cultural *H. rhamnoides* ssp. *sinensis*, 2n = 24; (**B12**) *E. lanceolata*, 2n = 28; (**B13**) *P. americana*, 2n = 24; (**B14**) *L. baviensis*, 2n = 24; (**B15**) *L. elongate*, 2n = 24; and (**B16**) *B. diaphana*, 2n = 28. Bar: 3 μm.

**Figure 3:**
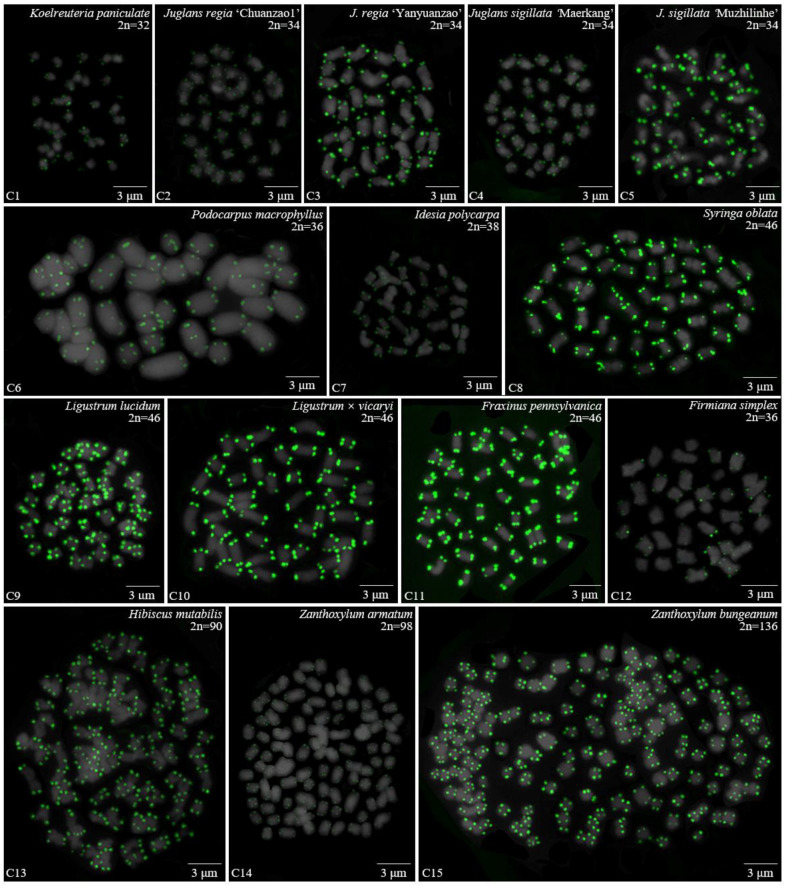
Oligo-FISH depicting the (AG_3_T_3_)_3_ present in 15 woody plants. All chromosomes in C1–C15 are from metaphase. Oligo-probe (AG_3_T_3_)_3_ is exhibited by green signals: (**C1**) *K. paniculata*, 2n = 32; (**C2**) *J. regia* ‘Chuanzao1’, 2n = 34; (**C3**) *J. regia* ‘Yanyuanzao’, 2n = 34; (**C4**) *J. sigillata* ‘Maerkang’, 2n = 34; (**C5**) *J. sigillata* ‘Muzhilinhe’, 2n = 34; (**C6**) *P. macrophyllus*, 2n = 36; (**C7**) *I. polycarpa*, 2n = 38; (**C8**) *S. oblata*, 2n = 46; (**C9**) *L. lucidum*, 2n = 46; (**C10**) *L.* × *vicaryi*, 2n = 46; (**C11**) *F. pennsylvanica*, 2n = 46; (**C12**) *F. simplex*, 2n = 36; (**C13**) *H. mutabilis*, 2n = 90; (**C14**) *Z. armatum*, 2n = 98; and (**C15**) *Z. bungeanum*, 2n = 136. Bar: 3 μm.

**Figure 4:**
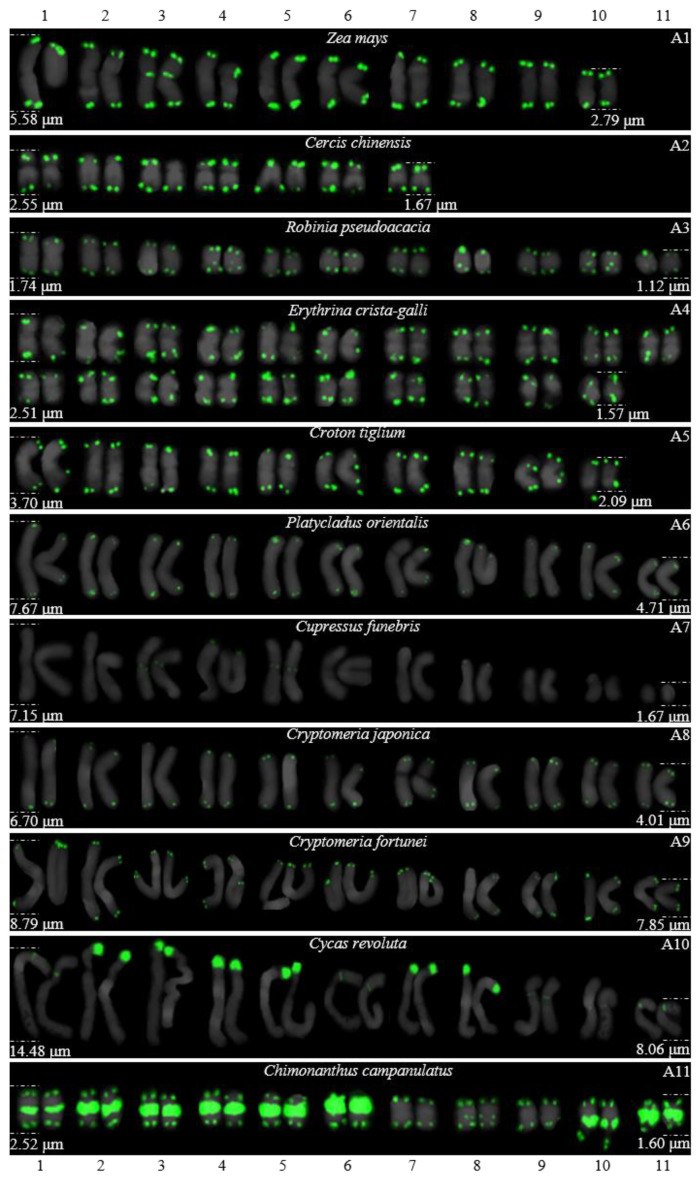
FISH karyograms of *Z. mays* and the ten woody plants from Figure 1. Oligo-probe (AG_3_T_3_)_3_ is exhibited by green signals. The upper/lower numbers (1–11) represent the chromosome pairs. The first/last chromosome of each plant were used to calculate the chromosome length: (**A1**) *Z. mays*, 2.79–5.58 μm; (**A2**) *C. chinensis*, 1.67–2.55 μm; (**A3**) *R. pseudoacacia*, 1.12–1.74 μm; (**A4**) *E. crista-galli*, 1.57–2.51 μm; (**A5**) *C. tiglium*, 2.09–3.70 μm; (**A6**) *P. orientalis*, 4.71–7.67 μm; (**A7**) *C. funebris*, 1.67–7.15 μm; (**A8**) *C. japonica*, 4.01–6.70 μm; (**A9**) *C. fortunei*, 7.85–8.79 μm; (**A10**) *C. revoluta*, 8.06–14.48 μm; and (**A11**) *C. campanulatus*, 1.60–2.52 μm.

**Figure 5:**
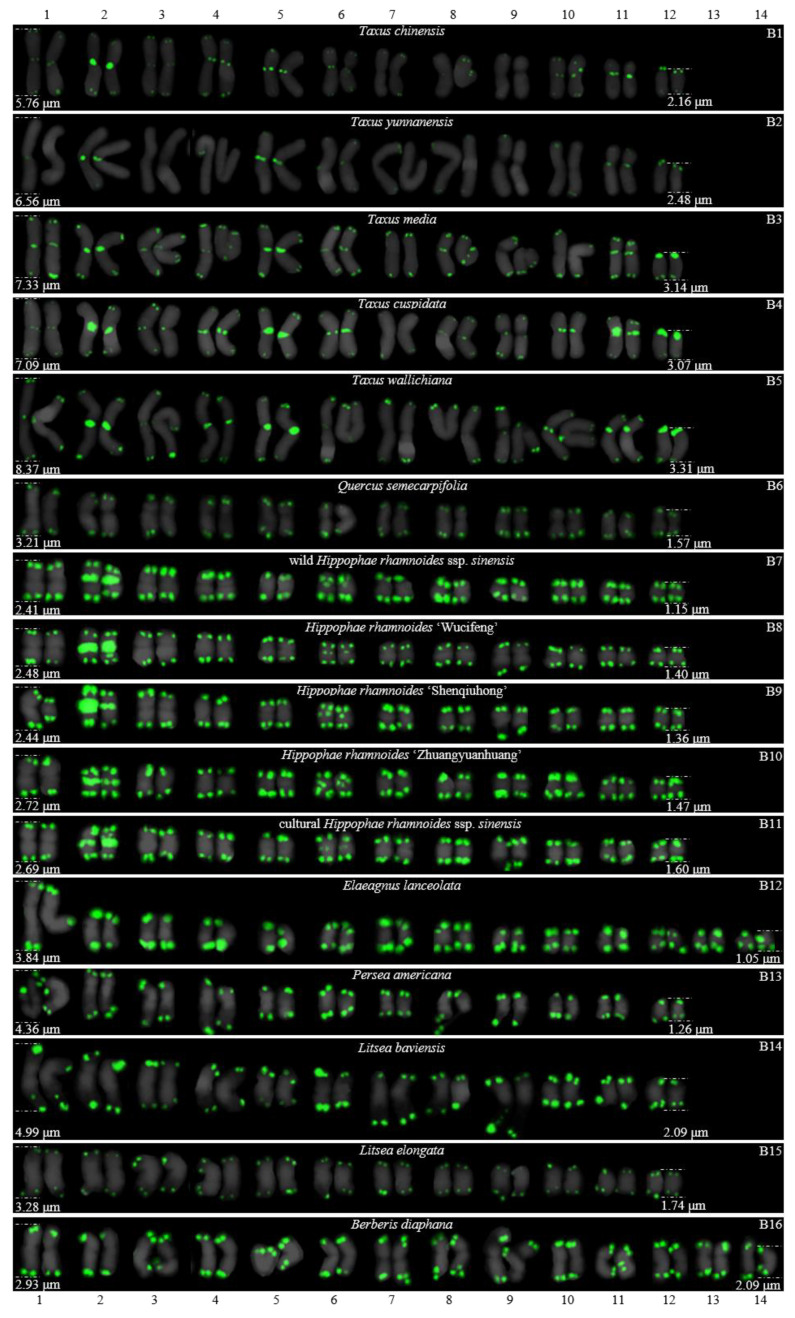
FISH karyograms of the 16 woody plants cutting from Figure 2. Oligo-probe (AG_3_T_3_)_3_ is exhibited by green signals. The upper/lower numbers (1–14) represent the chromosome pairs. The first/last chromosome of each plant were used to calculate the chromosome length: (**B1**) *T. chinensis*, 2.16–5.76 μm; (**B2**) *T. yunnanensis*, 2.48–6.56 μm; (**B3**) *T. media*, 3.14–7.33 μm; (**B4**) *T. cuspidata*, 3.07–7.09 μm; (**B5**) *T. wallichiana*, 3.31–8.37 μm; (**B6**) *Q. semecarpifolia*, 1.57–3.21 μm; (**B7**) wild *H. rhamnoides* ssp. *sinensis*, 1.15–2.41 μm; (**B8**) *H. rhamnoides* ‘Wucifeng’, 1.40–2.48 μm; (**B9**) *H. rhamnoides* ‘Shenqiuhong’, 1.36–2.44 μm; (**B10**) *H. rhamnoides* ‘Zhuangyuanhuang’, 1.47–2.72 μm; (**B11**) cultural *H. rhamnoides* ssp. *sinensis*, 1.60–2.69 μm; (**B12**) *E. lanceolata*, 1.05–3.84 μm; (**B13**) *P. americana*, 1.26–4.36 μm; (**B14**) *L. baviensis*, 2.09–4.99 μm; (**B15**) *L. elongate*, 1.74–3.28 μm; and (**B16**) *B. diaphana*, 2.09–2.93 μm.

**Figure 6:**
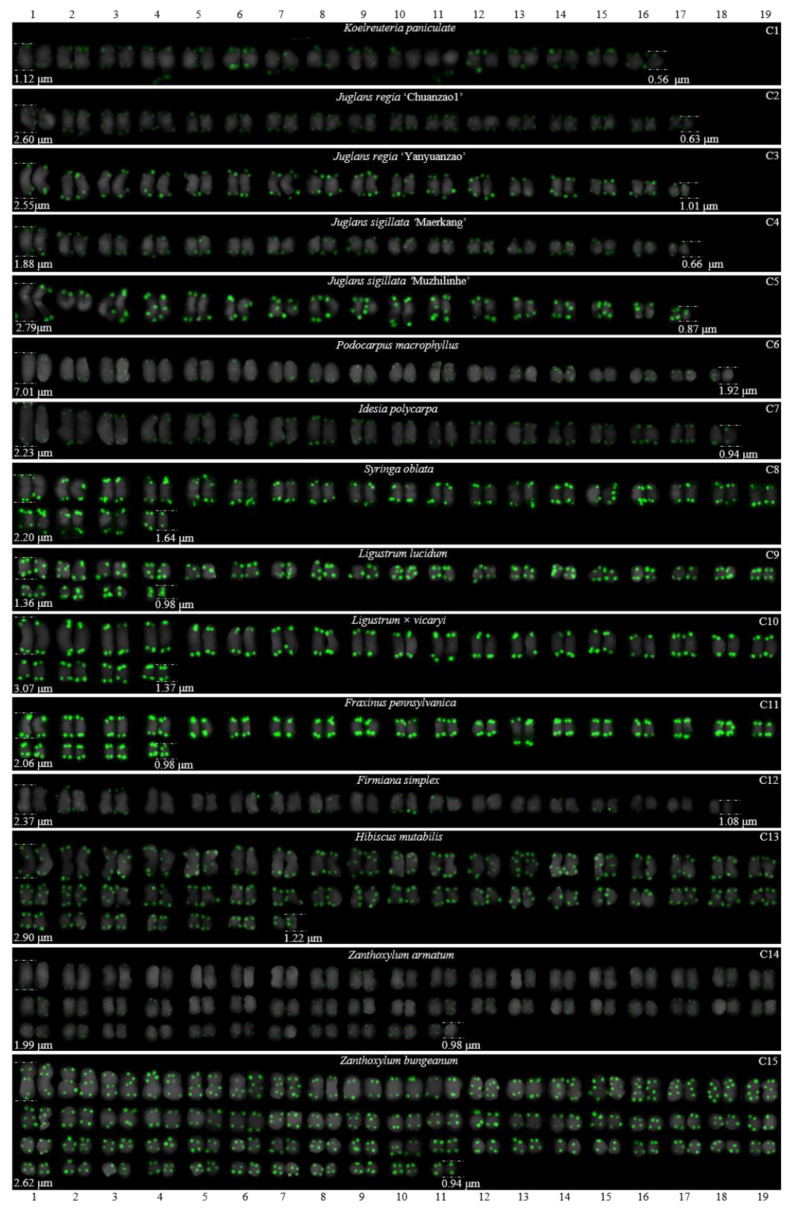
FISH karyogram of 15 woody plants cutting from Figure 3. Oligo-probe (AG_3_T_3_)_3_ is exhibited by green signals. The upper/lower numbers (1–19) represent the chromosome pairs. The first/last chromosome of each plant were used to calculate the chromosome length: (**C1**) *K. paniculata*, 0.56–1.12 μm; (**C2**) *J. regia* ‘Chuanzao1’, 0.63–2.60 μm; (**C3**) *J. regia* ‘Yanyuanzao’, 1.01–2.55 μm; (**C4**) *J. sigillata* ‘Maerkang’, 0.66–1.88 μm; (**C5**) *J. sigillata* ‘Muzhilinhe‘, 0.87–2.79 μm; (**C6**) *P. macrophyllus*, 1.92–7.01 μm; (**C7**) *I. polycarpa*, 0.94–2.23 μm; (**C8**) *S. oblata*, 1.64–2.20 μm; (**C9**) *L. lucidum*, 0.98–1.36 μm; (**C10**) *L.* × *vicaryi*, 1.37–3.07 μm; (**C11**) *F. pennsylvanica*, 0.98–2.06 μm; (**C12**) *F. simplex*, 1.08–2.37 μm; (**C13**) *H. mutabilis*, 1.22–2.90 μm; (**C14**) *Z. armatum*, 0.98–1.99 μm; and (**C15**) *Z. bungeanum*, 0.94–2.62 μm.

For 24 species and 14 families, this is the first time that (AG_3_T_3_)_3_ testing has been reported. To visualize the chromosomal distribution of (AG_3_T_3_)_3_ in *Z. mays* and the 41 woody plants, we performed FISH analysis, as shown in Figure 1(A1–A11), Figure 2(B1–B16), and Figure 3(C1–C15). To clearly display each chromosome distribution of the (AG_3_T_3_)_3_, we cut those in Figure 1, Figure 2 and Figure 3 and aligned them, as shown in Figure 4(A1–A11), Figure 5(B1–B16), and Figure 6(C1–C15).

The chromosome number and length for the considered species were sorted in Table 2. The chromosome number in the 42 plants ranged from 14 (*C. chinensis*, A3) to 136 (*Z. bungeanum*, C15). A total of 14 woody plants possessed 24 chromosomes (one third), whereas seven woody plants possessed 22 chromosomes (one sixth). The longest chromosome length of each plant ranged from 1.12 μm (*K. paniculata*, C1) to 14.48 μm (*C. revoluta*, A10), while the shortest chromosome length of each plant ranged from 0.56 μm (*K. paniculate*, C1) to 8.06 μm (*C. revoluta*, A10). A total of 23 woody plants (nearly one third) had chromosome length less than 3 μm, thus falling into the small chromosome category. Due to the indistinct location of centromeres and small size of chromosomes in many of the considered woody plants, further karyotype analysis—such as long/short arm length and karyotype formula—was not carried out. Karyotype asymmetry was assessed using the ratio of longest to shortest chromosome length. The largest ratio was 4.28 in *C. funebris* (A7), while the smallest ratio was 1.12 in *C. fortunei* (A9). The ratio for 17 plants ranged from 1 to 2 (40.5%), while that of 19 plants ranged from 2 to 3 (45.2%). The ratio was greater than 3 for six plants: *C. funebris* (A7), *E. lanceolata* (B12), *P. americana* (B13), *J. regia* ‘Chuanzao1’ (C2), *J. sigillata* ‘Muzhilinhe’ (C5), and *P. macrophyllus* (C6). These results indicated that abundant differences exist among 37 of the considered species. 

### 3.2. The Diverse Signal Patterns of (AG_3_T_3_)_3_ Reveal the Complex Genome Architecture

**Figure 7:**
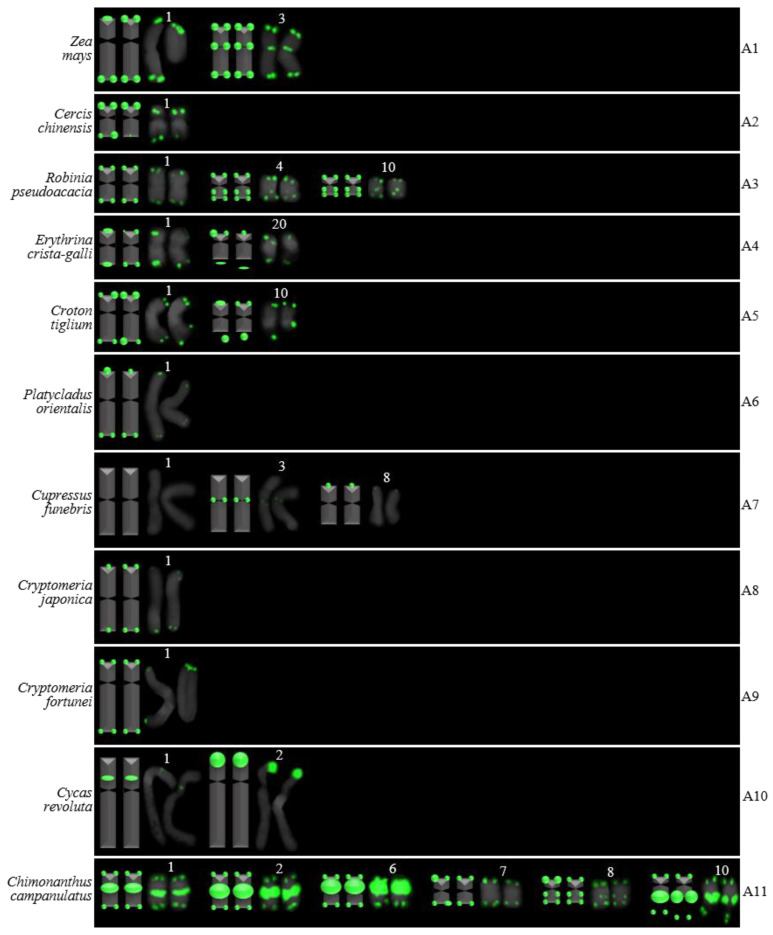
Ideograms of *Z. mays* and ten woody plants based on the signal patterns in the FISH karyograms shown in Figure 4: (**A1**) *Z. mays*; (**A2**) *C. chinensis*; (**A3**) *R. pseudoacacia*; (**A4**) *E. crista-galli*; (**A5**) *C. tiglium*; (**A6**) *P. orientalis*; (**A7**) *C. funebris*; (**A8**) *C. japonica*; (**A9**) *C. fortunei*; (**A10**) *C. revoluta*; and (**A11**) *C. campanulatus*. The numbers (1–4, 6–8, 10, 20) above the karyograms are the numbers of the chromosome pairs. Oligo-probe (AG_3_T_3_)_3_ is exhibited by green signals. Telomeric signals were observed at each chromosome terminus in (**A1**–**A6**,**A8**,**A9**,**A11**), while telomeric signals were absent at several chromosome termini in (**A7**,**A10**). Non-telomeric signals were observed at several chromosome termini in (**A1**,**A3**,**A7**,**A10**,**A11**). Telomeric signals deviated from the chromosome in (**A4**,**A5**,**A11**).

**Figure 8:**
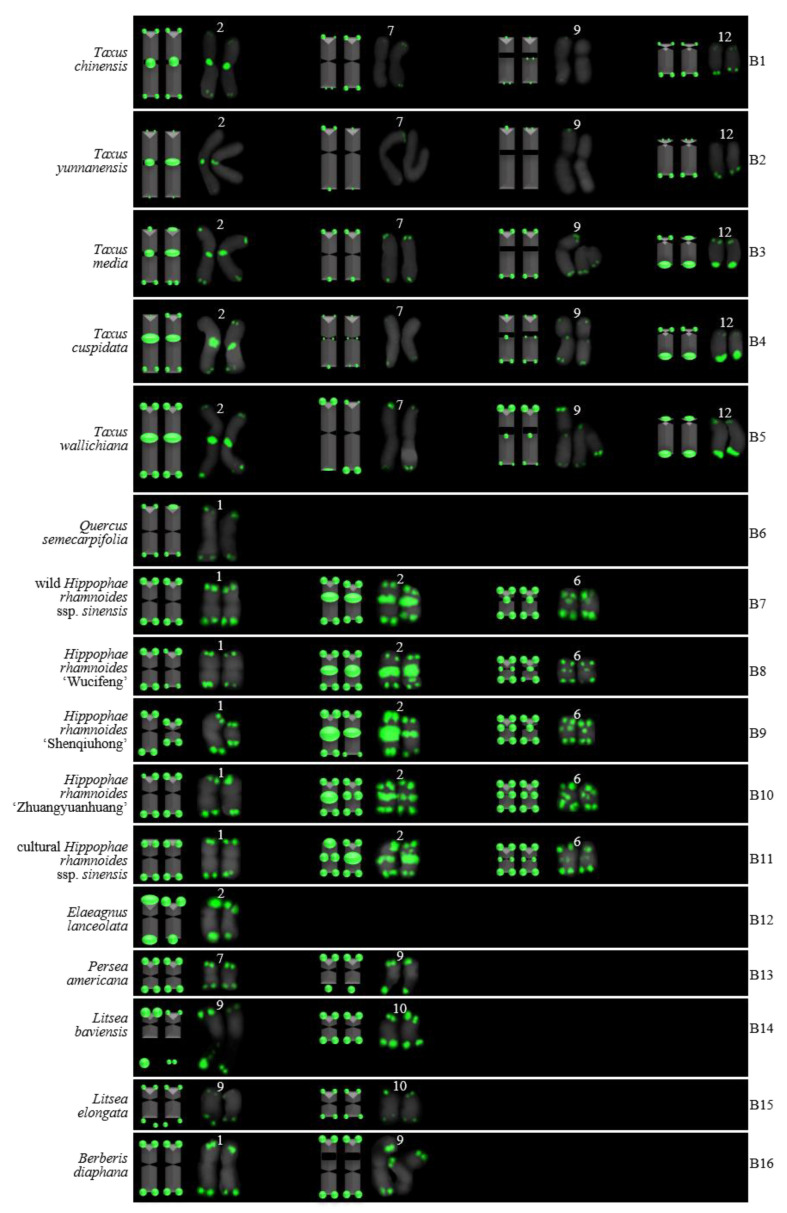
Ideograms of 16 woody plants based on the signal patterns in the FISH karyograms shown in Figure 5: (**B1**) *T. chinensis*; (**B2**) *T. yunnanensis*; (**B3**) *T. media*; (**B4**) *T. cuspidata*; (**B5**) *T. wallichiana*; (**B6**) *Q. semecarpifolia*; (**B7**) wild *H. rhamnoides* ssp. *sinensis*; (**B8**) *H. rhamnoides* ‘Wucifeng’; (**B9**) *H. rhamnoides* ‘Shenqiuhong’; (**B10**) *H. rhamnoides* ‘Zhuangyuanhuang’; (**B11**) cultural *H. rhamnoides* ssp. *sinensis*; (**B12**) *E. lanceolata*; (**B13**) *P. americana*; (**B14**) *L. baviensis*; (**B15**) *L. elongate*; and (**B16**) *B. diaphana*. The numbers (1, 2, 6, 7, 9, 10, 12) above the karyograms are the numbers of the chromosome pairs. Oligo-probe (AG_3_T_3_)_3_ is exhibited by green signals. Telomeric signals were observed at each chromosome terminus in (**B1**,**B3**–**B16**), while telomeric signals were absent at several chromosome termini in (**B2**). Non-telomeric signals were observed at several chromosome termini in (**B1**–**B5**,**B7**–**B11**). Telomeric signals deviated from the chromosome in (**B13**,**B14**).

**Figure 9:**
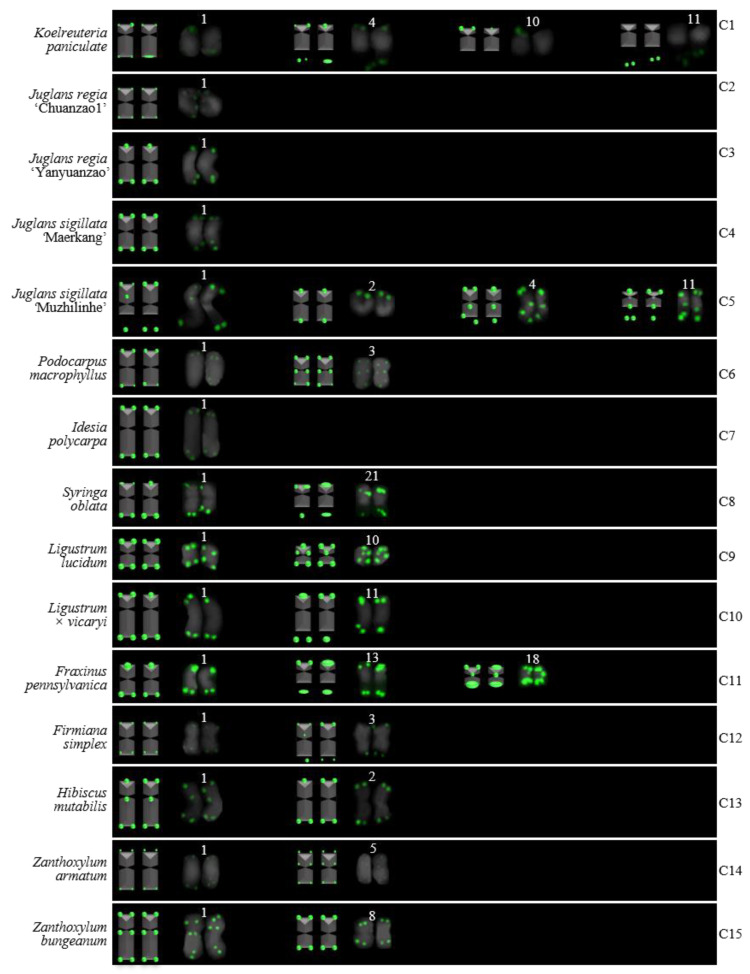
Ideograms of 15 woody plants based on the signal patterns in the FISH karyograms shown in Figure 6: (**C1**) *K. paniculata*; (**C2**) *J. regia* ‘Chuanzao1’; (**C3**) *J. regia* ‘Yanyuanzao’; (**C4**) *J. sigillata* ‘Maerkang’; (**C5**) *J. sigillata* ‘Muzhilinhe‘; (**C6**) *P. macrophyllus*; (**C7**) *I. polycarpa*; (**C8**) *S. oblata*; (**C9**) *L. lucidum*; (**C10**) *L.* × *vicaryi*; (**C11**) *F. pennsylvanica*; (**C12**) *F. simplex*; (**C13**) *H. mutabilis*; (**C14**) *Z. armatum*; and (**C15**) *Z. bungeanum*. The numbers (1–5, 8, 10, 11, 13, 18, 21) above the karyograms are the numbers of the chromosome pairs. Oligo-probe (AG_3_T_3_)_3_ is exhibited by green signals. Telomeric signals were observed at each chromosome terminus in (**C2**–**C15**), while telomeric signals were absent at several chromosome termini in C1. Non-telomeric signals were observed at several chromosome termini in (**C5**,**C6**,**C9**,**C11**–**C15**). Telomeric signals deviated from the chromosome in (**C1**,**C5**,**C8**,**C10**–**C12**).

To better investigate diversity of (AG_3_T_3_)_3_, different types of ideograms for the 42 plants were drawn based on the FISH karyograms shown in Figure 4, Figure 5 and Figure 6, which are illustrated in Figure 7, Figure 8 and Figure 9. Telomeric signals were observed at each chromosome terminus in 38 plants (90.5%, the first class): *Z. mays* (A1), *C. chinensis* (A2), *R. pseudoacacia* (A3), *E. crista-galli* (A4), *C. tiglium* (A5), *P. orientalis* (A6), *C. japonica* (A8), *C. fortune* (A9), *C. campanulatus* (A11), *T. chinensis* (B1), *T. media* (B3), *T. cuspidata* (B4), *T. wallichiana* (B5), *Q. semecarpifolia* (B6), wild *H. rhamnoides* ssp. *sinensis* (B7), *H. rhamnoides* ‘Wucifeng’ (B8), *H. rhamnoides* ‘Shenqiuhong’ (B9), *H. rhamnoides* ‘Zhuangyuanhuang’ (B10), cultural *H. rhamnoides* ssp. *sinensis* (B11), *E. lanceolata* (B12), *P. americana* (B13), *L. baviensis* (B14), *L. elongate* (B15), *B. diaphana* (B16), *J. regia* ‘Chuanzao1’ (C2), *J. regia* ‘Yanyuanzao’ (C3), *J. sigillata* ‘Maerkang’ (C4), *J. sigillata* ‘Muzhilinhe’ (C5), *P. macrophyllus* (C6), *I. polycarpa* (C7), *S. oblata* (C8), *L. lucidum* (C9), *L.* × *vicaryi* (C10), *F. pennsylvanica* (C11), *F. simplex* (C12), *H. mutabilis* (C13), *Z. armatum* (C14), and *Z. bungeanum* (C15). Meanwhile, telomeric signals were absent at several chromosome termini in only four woody plants (9.5%, the fourth class): *C. funebris* (A7), *C. revoluta* (A10), *T. yunnanensis* (B2), and *K. paniculata* (C1). Non-telomeric signals were observed at chromosome termini in 23 plants (54.8%, the second class): *Z. mays* (A1), *R. pseudoacacia* (A3), *C. funebris* (A7), *C. revoluta* (A10), and *C. campanulatus* (A11), *T. chinensis* (B1), *T. yunnanensis* (B2), *T. media* (B3), *T. cuspidata* (B4), *T. wallichiana* (B5), wild *H. rhamnoides* ssp. *sinensis* (B7), *H. rhamnoides* ‘Wucifeng’ (B8), *H. rhamnoides* ‘Shenqiuhong’ (B9), *H. rhamnoides* ‘Zhuangyuanhuang’ (B10), cultural *H. rhamnoides* ssp. *sinensis* (B11), *J. sigillata* ‘Muzhilinhe’ (C5), *P. macrophyllus* (C6), *L. lucidum* (C9), *F. pennsylvanica* (C11), *F. simplex* (C12), *H. mutabilis* (C13), *Z. armatum* (C14), and *Z. bungeanum* (C15). Telomeric signals outside of the chromosome were observed in 11 woody plants (26.2%, the third class): *E. crista-galli* (A4), *C. tiglium* (A5), *C. campanulatus* (A11), *P. americana* (B13), *L. baviensis* (B14), *K. paniculata* (C1), *J. sigillata* ‘Muzhilinhe’ (C5), *S. oblata* (C8), *L.* × *vicaryi* (C10), *F. pennsylvanica* (C11), and *F. simplex* (C12). 

**Figure 10:**
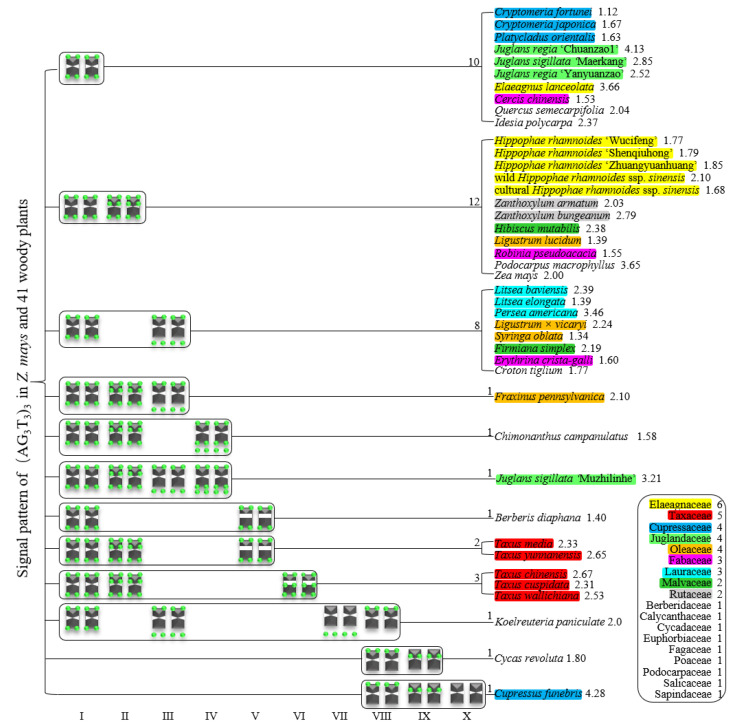
Signal pattern of (AG_3_T_3_)_3_ in *Z. mays* and 41 woody plants. Signal patterns type I–X are summarized based on Figure 7, Figure 8 and Figure 9. The number between the signal pattern and the species represents the type/type combination, including the species number. The number after the species represents the ratio of longest to shortest chromosome length. Elaeagnaceae includes six woody plants (yellow), Taxaceae includes five woody plants (red), Cupressaceae includes four woody plants (dark blue), Juglandaceae includes four woody plants (light green), Oleaceae includes four woody plants (orange), Fabaceae includes three woody plants (pink), Lauraceae includes three woody plants (light blue), Malvaceae includes two woody plants (dark green), Rutaceae includes two woody plants (grey), and Berberidaceae, Calycanthaceae, Cycadaceae, Euphorbiaceae, Fagaceae, Poaceae, Podocarpaceae, Salicaceae, and Sapindaceae each include one woody plant, respectively.

Furthermore, we summarized the results shown in Figure 7, Figure 8 and Figure 9 in order to produce the (AG_3_T_3_)_3_ signal pattern presented in Figure 10. The results for *Z. mays* and 41 woody plants, belonging to 18 families, are shown, including six plants in Elaeagnaceae (yellow), five in Taxaceae (red), four in Cupressaceae (dark blue), four in Juglandaceae (light green), four in Oleaceae (orange), three in Fabaceae (pink), three in Lauraceae (light blue), two in Malvaceae (dark green), two in Rutaceae (grey), and one in each of Berberidaceae, Calycanthaceae, Cycadaceae, Euphorbiaceae, Fagaceae, Poaceae, Podocarpaceae, Salicaceae, and Sapindaceae, respectively. Except for Lauraceae and Rutaceae, which each presented a single signal pattern type, the other families (Elaeagnaceae, Cupressaceae, Juglandaceae, Oleaceae, Lauraceae, and Malvaceae) all presented at least two signal pattern types. 

As shown in Figure 10, there were ten (AG_3_T_3_)_3_ signal pattern types in total: Type I, chromosome only includes signal at both ends; Type II, chromosome not only includes signal at both ends but also includes a non-telomeric signal location; Type III, chromosome includes single end signal, and the other telomeric signals outside of the chromosome; Type IV, chromosome not only includes signal at both ends, but also includes telomeric signal deviating from chromosome; Type V, chromosome not only includes signal at both ends, but also includes a large primary constriction; Type VI: chromosome not only includes signal at both ends signal, but also includes a large primary constriction, as well as a non-telomeric signal location; Type VII, chromosome only includes telomeric signal outside of the chromosome; Type VIII, chromosome only includes single end signal; Type IX, chromosome only includes non-telomeric signal location; and Type X, chromosome includes no signals. These types of signal pattern indicate that there is an abundant diversity in (AG_3_T_3_)_3_ signal arrangement.

All 42 plants possessed the 12 signal pattern types or type combinations shown in Figure 10. Ten woody plants only possessed signal pattern type I; *Z. mays* and 11 woody plants possessed the combination of type I + type II; eight woody plants possessed the combination of type I + type III; *P. macrophyllus* possessed the combination of type I + type II + type III; *C. campanulatus* possessed the combination of type I + type II + type IV; *J. sigillata* ‘Muzhilinhe’ possessed the combination of type I + type II + type III + type IV; *B. diaphana* possessed the combination of type I + type V; *T. media* and *T. yunnanensis* possessed the combination of type I + type II + type V; *T. chinensis*, *T. cuspidata*, and *T. wallichiana* possessed the combination of type I + type II + type VI; *K. paniculate* possessed the combination of type I + type III + type VII + type VIII; *C. revolute* possessed the combination of type VIII + type IX; and *C. funebris* possessed the combination of type VIII + type IX + type X. 

There were diverse signal patterns of (AG_3_T_3_)_3_ among 37 species, indicating a complex genome architecture. For example, considering (i) Elaeagnaceae, five plants of *H. rhamnoides* possessed type I + type II, but *E. lanceolata* possessed type I; (ii) in Taxaceae, *T. media* and *T. yunnanensis* possessed the combination type I + type II + type V, but *T. chinensis*, *T. cuspidata*, and *T. wallichiana* possessed the combination type I + type II + type VI; (iii) in Cupressaceae, *C. fortune*, *C. japonica,* and *P. orientalis* possessed type I, but *C. funebris* possessed the combination type VIII + type IX + type X; (iv) in Juglandaceae, *J. regia* ‘Chuanzao1’, *J. regia* ‘Yanyuanzao’, and *J. sigillata* ‘Maerkang’ possessed type I, but *J. sigillata* ‘Muzhilinhe’ possessed the combination type I + type II + type III + type IV; (v) in Oleaceae, *L. lucidum* possessed the combination type I + type II, *L.* × *vicaryi* and *S. oblata* possessed the combination type I + type III, and *F. pennsylvanica* possessed the combination type I + type II + type III; (vi) in Fabaceae, *C. chinensis* possessed type I, *R. pseudoacacia* possessed the combination type I + type II, and *E. crista-galli* possessed the combination type I + type III; and (vii) in Malvaceae, *H. mutabilis* possessed the combination type I + type II, but *F. simplex* possessed the combination type I + type III. 

The number shown after the species name in Figure 10 represents the ratio of longest to shortest chromosome length, indicating karyotype asymmetry. Type I included ten plants with ratio ranging from 1.12–4.13, with variance of 0.94. Type I + Type II included 12 plants with ratio ranging from 1.39–3.65, with variance of 0.38. Type I + Type II + Type III included eight plants with ratio ranging from 1.34–3.46, with variance of 0.48. These results indicate that chromosomes with conserved telomeric signal (Type I), in fact, have a wider range of karyotype asymmetry (VAR 0.94), while chromosomes with non-telomeric signals (Type II, III) have relatively concentrated karyotype asymmetry (VAR 0.38 and 0.48, respectively). In addition, no correlation between non-telomeric signals and karyotype asymmetry was observed.

### 3.3. Proposed Origin of (AG_3_T_3_)_3_ Signal Diversity

**Figure 11:**
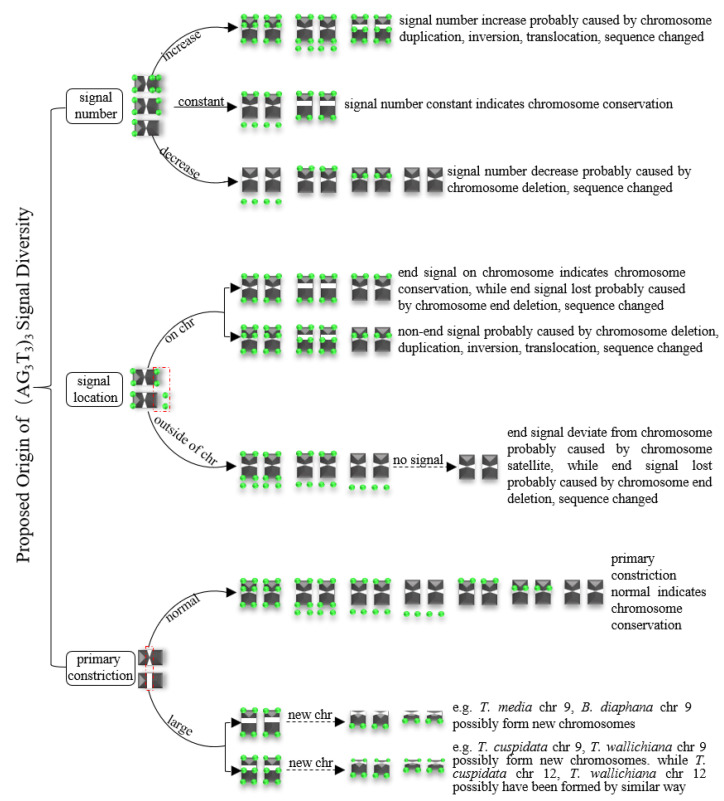
Proposed origin of (AG_3_T_3_)_3_ signal diversity. The variations in signal number and signal location were likely caused by chromosome deletion, duplication, inversion, translocation, as sequence changes, as well as chromosome satellites, while primary constriction became large due to chromosome breakage and the formation of new chromosomes.

Based on Figure 7, Figure 8, Figure 9 and Figure 10, the proposed origin of (AG_3_T_3_)_3_ signal diversity is illustrated in Figure 11. There are three major groups. (i) Signal number: Increase signal number is likely caused by chromosome duplication, inversion, translocation, and/or sequence changes; a constant signal number indicates chromosome conservation and a decreased signal number is likely caused by chromosome deletion and/or sequence changes. (ii) Signal location: End signals on chromosomes indicate chromosome conservation; non-end signals are likely caused by chromosome deletion, duplication, inversion, translocation, and/or sequence changes; end signals deviating from the chromosome are probably caused by chromosome satellites, while end signal loss is probably caused by chromosome end deletion and/or sequence changes. (iii) Primary constriction: Normal primary constriction indicates chromosome conservation, while primary constriction likely becomes large due to chromosome breakage and the formation of new chromosomes (e.g., in Figure 8, *T. media* chromosome 9 and *B. diaphana* chromosome 9, *T. cuspidata* chromosome 9 and *T. wallichiana* chromosome 9 possibly indicate the formation of new chromosomes, while *T. cuspidata* chromosome 12 and *T. wallichiana* chromosome 12 were possibly formed in a reverse manner). 

In brief, the variations in signal number and signal location were probably caused by chromosome deletion, duplication, inversion, translocation, and sequence changes, as well as chromosome satellites. It is likely that large primary constriction was due to chromosome breakage and the formation of new chromosomes. 

## 4. Discussion

### 4.1. Karyotype Analysis of Z. mays and 41 Woody Plants

Chromosome number, size, centromere location, long/short arm ratio, and satellites are basic characteristics of karyotype. The presence of telomeric (AG_3_T_3_)_3_ in both chromosome ends may guarantee the accuracy of chromosome counting. The chromosome number in the 42 plants ranged from 14 (*C. chinensis*) to 136 (*Z. bungeanum*). There were six woody plants that presented chromosome numbers different to that reported in previous studies: *J. regia* ‘Chuanzao1’, *J. regia* ‘Yanyuanzao’, *J. sigillata* ‘Maerkang’, and *J. sigillata* ‘Muzhilinhe’ presented 2n = 34, a result supported by the work of Luo and Chen [48] but contradicted by Mu and Xi [54] and Mu et al. [55] (who reported 2n = 32). The differences were probably caused by hybridization, aneuploidization/among-population variation, or inaccurate chromosome number counts. *P. macrophyllus* presented 2n = 36, a result supported by the work of Hizume et al. [56] but contradicted by Zhu et al. [57] for eight small chromosomes, which were treated as satellite chromosomes in the latter. *Z. armatum* ‘Jinyang Qinghuajiao’ presented 2n = 98 in this study, which is contradicted by the work of Luo et al. [58], who reported 2n = ~128 for another variety, *Z. armatum* ‘Hanyuan Putao Qingjiao’. After excluding the experimental count error, we infer this big difference to have been caused by the confusion and complexity between *Z. armatum* varieties. We tested 16 *Z. armatum* varieties by FISH and SSR in another study in order to address this issue. Another reason why the chromosome numbers differed from previous studies is because of the limited available research on the chromosome numbers of woody plants. Compared to herbaceous plants, it is more difficult to obtain their chromosome preparation due to root lignification.

The chromosome lengths of the 42 plants in this study ranged from 0.56 μm (*K. paniculate*) to 14.48 μm (*C. revoluta*), while the ratio of longest chromosome to shortest chromosome ranged from 1.12 (*K. paniculate*) to 4.28 (*C. revoluta*), both of which indicate large differences among the considered species. Previous studies have reported the chromosome sizes of 12 species: *R. pseudoacacia* presented a size of 1.12–1.74 μm, which was close to that of He et al. [51] (0.94–1.67 μm); *H. mutabilis* presented 1.22–2.90 μm, close to that of Luo and He [47] (1.18–3.0 μm); *B. diaphana* presented 2.09–2.93 μm, close to that of Liu and Luo [50] (1.82–2.85 μm); *F. pennsylvanica* presented 0.98–2.06 μm, close to that of Luo and Liu [49] (1.12–2.06 μm); *H. rhamnoide* presented 1.15–2.72 μm, close to that of Xing et al. [59] (0.97–2.77 μm), but varying widely from that of Liu and Sheng [60] (1.67–4.44 μm); *C. campanulatus* presented 1.60–2.52 μm, differing from that of Luo and Chen [52] (1.07–2.41 μm); *Z. armatum* presented 0.98–1.99 μm, differing from that of Luo et al. [58] (1.23–2.34 μm); *S. oblata* presented 1.64–2.20 μm, differing from that of Luo and Liu [49] (1.25–2.32 μm); *L. lucidum* presented 0.98–1.36 μm, differing from that of Luo and Liu [49] (1.05–1.85 μm); *L.* × *vicaryi* presented 1.37–3.07 μm, differing from that of Luo and Liu [49] (1.25–2.83 μm); *J. regia* presented 0.63–2.60 μm, differing from that of Luo and Chen [48] (0.97–2.16 μm); finally, *J. sigillata* presented 0.66–2.79 μm, differing from that of Luo and Chen [48] (0.98–2.65 μm). Hence, chromosome length was more suitable for using in qualitative analysis than quantitative analysis.

A total of 23 woody plants (nearly one third) had chromosome lengths less than 3 μm, thus belonging to the small chromosome group. Due to the indistinct location of the centromere and the small size of chromosomes in many of the woody plants, as well as the chromosome length fluctuating according to the chromosome division phase and measuring tool, further karyotype analysis (e.g., long/short arm length and karyotype formula) was not carried out. Chromosome length is also not discussed further.

### 4.2. Occurrence of (AG_3_T_3_)_3_ in Woody Plants

AG_3_T_3_ is a telomeric repetitive tandem that is conserved in higher plant chromosome telomeres. This oligos is included in a B chromosome-specific sequence, named M8-2D, in *Z. mays* [53]. M8-2D has shown low homology to sequences from the chromosome 4 centromere in *Z. mays* but has been detected in B chromosome centromeric and telomeric regions in *Z. mays*. Nonetheless, (AG_3_T_3_)_3_ had not been previously tested in *Z. mays* before this study. 

Overall, (AG_3_T_3_)_3_ was found to occur in *Z. mays* and 41 woody plants, belonging to 37 species, 27 genera, and 18 families. Previous research has reported the occurrence of (AG_3_T_3_)_3_ in 14 woody species, including *A. fruticose* [51], *B. soulieana* [50], *B. diaphana* [50], *C. campanulatus* [52], *F. pennsylvanica* [49], *H. mutabilis* [47]), *H. rhamnoides* [11], *J. regia* [48], *J. sigillata* [48], *L. lucidum* [49], *L. × vicaryi* [49], *R. pseudoacacia* [51], *S. oblata* [49], and *S. japonicum* [51].

The *Arabidopsis*-type telomeric repeat sequences T_3_AG_3_ are most like AG_3_T_3_, which are still the most frequent even in woody plants—such as species of *Abies* [37], *Aralia* [41], *Cycas* [39], *Dendropanax* [41], *Eleutherococcus* [41], *Kalopanax* [41], *Malus* [23], *Picea* [23], *Pinus* [23,40,42,43,44], *Populus* [35], *Podocarpus* [46], and *Zamia* [23,61]—but has been shown to be absent in species of *Cestrum*, *Sessea*, and *Vestia* [14]. Nevertheless, these sequences cannot be entirely equated, due to the existence of at least ten variable telomere sequences (T_x_A_y_G_z_)_n_—including TAG_3_ [4], TA_2_G_2_ [5], T_2_AG_2_ [6], T_2_AG_3_ [7], TA_2_G_3_ [5], T_3_AG_3_ [8,9,10], T_4_AG_3_ [12], A_2_TG_6_ [13], T_2_AT_2_AG_3_ [7], and T_6_AG_3_ [14,15]—and at least seven variants, such as TCAG_2_ [16], T_2_AG_2_C [17], T_2_CAG_2_ [18], T_3_CAG_2_ [18], C_3_TA_3_ [19], T_2_N_4_AG_3_ [20], and CTCG_2_T_2_ATG_3_ [21]. 

### 4.3. The Chromosomally Diverse Distribution of (AG_3_T_3_)_3_ Reveals the Complex Genome Architecture of Woody Plants

The (AG_3_T_3_)_3_ distribution is mostly conserved in the chromosome termini of woody plants. (AG_3_T_3_)_3_ has only been detected at all chromosome termini in *B. soulieana*, *B. diaphana* [50], *F. pennsylvanica* [49], *H. mutabilis* [47], *J. regia* [48], *J. sigillata* [48], *L. lucidum* [49], *L. × vicaryi* [49], and *S. oblata* [49]. The location of (AG_3_T_3_)_3_ at chromosome termini may indicate the integrality of the chromosome, thus ensuring the accuracy of chromosome counting. Occasionally, (AG_3_T_3_)_3_ has been detected not only at chromosome termini, but also as intercalary bands in *A. fruticose*, *R. pseudoacacia*, *S. japonicum* [51], *C. campanulatus* [52], and *H. rhamnoides* [11]. In *A. fruticose*, *R. pseudoacacia*, and *S. japonicum,* only two weak non-telomeric signals have been described, while *H. rhamnoides* presented two very strong, two clear, and another two weak non-telomeric signals in six chromosomes. Furthermore, (AG_3_T_3_)_3_ has shown exceptionally high levels of diversity in *C. campanulatus*, with signal intensity from weak to very strong, signals located outside the chromosome (satellites), and signals located at chromosome termini, sub-telomeric regions, and proximal regions. All 22 chromosomes presented telomeric signals, while 16 chromosomes presented non-telomeric signals in *C. campanulatus* [52]. (AG_3_T_3_)_3_ in intercalary sites may be used as informative markers to distinguish woody plants. The telomeric and non-telomeric (AG_3_T_3_)_3_ reveal the complex genome architecture of woody plants.

In this study of 42 plants, the first class (90.5% of plants) presented telomeric signals at each chromosome terminus; this result is supported by previous studies [11,47,48,49,50,51,52]. Only four woody plants presented telomeric signal absence at several chromosome termini (*C. funebris*, *C. revoluta*, *K. paniculata*, and *T. yunnanensis*). The probable reasons for the absence of signals were: (i) Telomeric signal occurrence which was lost during experiment; and (ii) changed end sequences, such that the ends presented no (AG_3_T_3_)_3_ signal. The second class (54.8% of plants) presented non-telomeric signal at several chromosome locations. *C. campanulatus*, *H. rhamnoides*, and *R. pseudoacacia* had non-telomeric signals, as supported by Luo and Chen [52], Luo et al. [11], and He et al. [51]. However, *F. pennsylvanica*, *H. mutabilis*, *J. sigillata* ‘Muzhilinhe‘, and *L. lucidum* presented non-telomeric signals in this study, but only showed telomeric signals in previous research [47,48,49]. The probable reasons for such discrepant signals were: (i) non-telomeric signal occurrence was lost during the previous experiments; and (ii) the variation in different batches of materials in the same species (i.e., intraspecific variation). Previous evidence suggested several species with a chromosome number of n = 21 or higher may present interstitial telomeric sequences [62]. In the present work, five species, *F. pennsylvanica* (2n = 46), *L.* × *vicaryi* (2n = 46), *H. mutabilis* (2n = 90), *Z. armatum* (2n = 98), and *Z. bungeanum* (2n = 136), presented interstitial telomeric signals, all having a chromosome number 2n > 42. It is possible that high chromosome number species have not been used as frequently in previous studies. The third class (26.2% of plants) presented telomeric signal deviating from the chromosome. *C. campanulatus*, *F. pennsylvanica*, L. × *vicaryi*, and *S. oblata* showed this type of signal, as supported by the results of Luo and Liu [49] and Luo and Chen [52]. *J. sigillata* ‘Muzhilinhe’ also presented this type of signal, in contrast to the results of Luo and Chen [48]. The probable reasons for this discrepancy are similar to those for the second class. 

Unusual telomere sequences described by non-telomeric signals are, in many cases, connected with high C-values [63]; for example, in species of *Cestrum* and *Allium* [64]. In the present study, *Z. mays*, *Z. armatum*, and *Z. bungeanum* presented non-telomeric signals along with giant C-values (*Zea,* 3.8 pg; *Zanthoxylum,* 4.57 pg) [65] (https://cvalues.science.kew.org/, accessed on 17 May 2022). Nevertheless, the small C-values of *Juglans* (0.64 pg), *Robinia* (0.74 pg), and *Chimonanthus* (0.86 pg) were also accompanied by non-telomeric signals (*C.* *campanulatus*, *J. sigillata* ‘Muzhilinhe‘, *R. pseudoacacia*); especially for *C.* *campanulatus*, which presented highly diverse non-telomeric signals. Hence, to the best of our knowledge, there is no correlation between the presence of non-telomeric signals and the C-value in woody plants; this result agrees with the conclusion of Gorelick et al. [66].

### 4.4. Proposed Origin of (AG_3_T_3_)_3_ Diversity in Woody Plants

Telomeric sequences are not found exclusively at chromosome termini, but also in non-terminal sites of chromosomes in many species [23,24,25]. Interstitial telomeric sequences may represent a significant part of telomeric DNA. Bolzán [3] has revealed two major types of interstitial telomeric sequences: one is heterochromatic and is largely observed in centromeric or pericentromeric regions (e.g., in this study, *C.* *campanulatus,* five species of *Taxus,* and five plants of *H. rhamnoides*). The other type is short and distributed at various sites throughout chromosomes, such as in *J. sigillata* ‘Muzhilinhe’ and *R. pseudoacacia*. 

Interstitial telomeric sequences could be considered the result of chromosomal rearrangements [23,24,67,68]. Messier et al. [69] explained interstitial telomeric sequences through the creation of a small number of repeats by random mutations followed by repeat expansion towards two flanks. In the present work, the non-terminal (AG_3_T_3_)_3_ signals were likely caused by chromosome deletion, duplication, inversion, translocation, and sequence changes, all of which are chromosomal reorganizations. Previous researchers have hypothesized that interstitial telomeric sequences in the heterochromatic region could be the trace of the chromosome end fusion, causing descendent hypoploidy (a decrease in the chromosome number) [28,29,30]. In the present work, we observed large primary constriction in chromosome 9 of *B. diaphana* and five species of *Taxus*. We may infer that these chromosomes with large primary constriction will possibly lead to ascent hyperploidyc. Thus, in the case of large primary constriction with non-end (AG_3_T_3_)_3_ signal, such as *T. cuspidate* chromosome 9 or *T. wallichiana* chromosome 9, ascent hyperploidy may occur. Similarly, we may also infer that the telocentric chromosome 12 in five species of *Taxus* was possibly formed in a reverse manner. However, in the case of large primary constriction with no signal, such as that observed in *T. media* chromosome 9 and *B. diaphana* chromosome 9, chromosome breakage is likely unrelated to interstitial (AG_3_T_3_)_3_.

## 5. Conclusions

In this paper, we examined *Z. mays* and 41 woody plants, established FISH physical mapping, described the diverse distribution of (AG_3_T_3_)_3_, and disclosed the complex genome architecture of woody plants. We inferred that the observed non-telomeric signals were probably caused by chromosome arrangements. We intend to continue our research by testing more woody plants, such as species of Calycanthaceae, to explore the abundant non-telomeric (AG_3_T_3_)_3_, as well as Rutaceae, to determine the chromosome number. 

## Figures and Tables

**Table 1:** Details of *Z. mays* and the 41 woody plants used in this study.

Family	No.	Species	Collection Location
Poaceae	1	*Z. mays* L.	Wenjiang, Sichuan
Fabaceae	2	*Cercis chinensis* Bunge	Wenjiang, Sichuan
	3	*R. pseudoacacia* L.	Wenjiang, Sichuan
	4	*Erythrina crista-galli* L.	Wenjiang, Sichuan
Euphorbiaceae	5	*Croton tiglium* L.	Yaan, Sichuan
Cupressaceae	6	*Platycladus orientalis* (L.) Franco	Wenjiang, Sichuan
	7	*Cupressus funebris* Endl.	Wenjiang, Sichuan
	8	*Cryptomeria japonica* (L. f.) D. Don	Wenjiang, Sichuan
	9	*Cryptomeria fortune* Hooibrenk ex Otto et Dietr.	Wenjiang, Sichuan
Cycadaceae	10	*Cycas revolute* Thunb.	Wenjiang, Sichuan
Calycanthaceae	11	*C. campanulatus* R.H.Chang, C.S.Ding	Jinniu, Sichuan
Taxaceae	12	*Taxus chinensis* (Pilger) Rehd.	Wenchuan, Sichuan
	13	*Taxus yunnanensis* W.C.Cheng and L.K.Fu	Yanan, Sichuan
	14	*Taxus media*	Yanan, Sichuan
	15	*Taxus cuspidate* Sieb. et Zucc.	Dujiangyan, Sichuan
	16	*Taxus wallichiana* Zucc.	Yanan, Sichuan
Fagaceae	17	*Quercus semecarpifolia* Smith	Wenchuan, Sichuan
Elaeagnaceae	18	wild *H. rhamnoides* ssp. *sinensis* Rousi	Wenchuan, Sichuan
	19	*H. rhamnoides* L. ‘Wucifeng’	Wenchuan, Sichuan
	20	*H. rhamnoides* L. ‘Shenqiuhong’	Wenchuan, Sichuan
	21	*H. rhamnoides* L. ‘Zhuangyuanhuang’	Wenchuan, Sichuan
	22	cultural *H. rhamnoides* ssp. *sinensis* Rousi	Wenchuan, Sichuan
	23	*Elaeagnus lanceolate* Warb. apud Diels	Wenchuan, Sichuan
Lauraceae	24	*Persea Americana* Mill	Xichang, Sichuan
	25	*Litsea baviensis* Lec.	Jinniu, Sichuan
	26	*Litsea elongate* Lec.	Jinniu, Sichuan
Berberidaceae	27	*B. diaphana* Maxim.	Wenchuan, Sichuan
Sapindaceae	28	*Koelreuteria paniculate* Laxm.	Wenjiang, Sichuan
Juglandaceae	29	*J. regia* L. ‘Chuanzao1’	Qingbaijiang, Sichuan
	30	*J. regia* L. ‘Yanyuanzao’	Yanyuan, Sichuan
	31	*J. sigillata* Dode ‘Maerkang’	Maerkang, Sichuan
	32	*J. sigillata* Dode ‘Muzhilinhe’	Gulin, Sichuan
Podocarpaceae	33	*Podocarpus macrophyllus* (Thunb.) Sweet	Wenjiang, Sichuan
Salicaceae	34	*Idesia polycarpa* Maxim.	Wenjiang, Sichuan
Oleaceae	35	*S. oblata* Lindl.	Wenjiang, Sichuan
	36	*L. lucidum* Ait.	Wenjiang, Sichuan
	37	*L. × vicaryi* Rehder	Wenjiang, Sichuan
	38	*F. pennsylvanica* Marsh.	Wenjiang, Sichuan
Malvaceae	39	*Firmiana simplex* (Linnaeus) W. Wight	Wenjiang Sichuan
	40	*H. mutabilis* L.	Jinniu, Sichuan
Rutaceae	41	*Zanthoxylum armatum* DC.	Jinyang, Sichuan
	42	*Zanthoxylum bungeanum* Maxim.	Hanyuan, Sichuan

**Table 2:** Chromosome number and length of the 42 plants used in this study.

Accession	Species	Chromosome Number	Chromosome Length	Karyotype Asymmetry
A1	*Z. mays*	2n = 20	2.79–5.58 μm	2.00
A2	*C. chinensis*	2n = 14	1.67–2.55 μm	1.53
A3	*R. pseudoacacia*	2n = 22	1.12–1.74 μm	1.56
A4	*E. crista-galli*	2n = 42	1.57–2.51 μm	1.60
A5	*C. tiglium*	2n = 20	2.09–3.70 μm	1.77
A6	*P. orientalis*	2n = 22	4.71–7.67 μm	1.63
A7	*C. funebris*	2n = 22	1.67–7.15 μm	4.28
A8	*C. japonica*	2n = 22	4.01–6.70 μm	1.67
A9	*C. fortune*	2n = 22	7.85–8.79 μm	1.12
A10	*C. revolute*	2n = 22	8.06–14.48 μm	1.80
A11	*C. campanulatus*	2n = 22	1.60–2.52 μm	1.58
B1	*T. chinensis*	2n = 24	2.16–5.76 μm	2.68
B2	*T. yunnanensis*	2n = 24	2.48–6.56 μm	2.65
B3	*T. media*	2n = 24	3.14–7.33 μm	2.33
B4	*T. cuspidate*	2n = 24	3.07–7.09 μm	2.31
B5	*T. wallichiana*	2n = 24	3.31–8.37 μm	2.53
B6	*Q. semecarpifolia*	2n = 24	1.57–3.21 μm	2.04
B7	wild *H. rhamnoides* ssp. *sinensis*	2n = 24	1.15–2.41 μm	2.10
B8	*H. rhamnoides* L. ‘Wucifeng’	2n = 24	1.40–2.48 μm	1.77
B9	*H. rhamnoides* L. ‘Shenqiuhong’	2n = 24	1.36–2.44 μm	1.79
B10	*H. rhamnoides* L. ‘Zhuangyuanhuang’	2n = 24	1.47–2.72 μm	1.84
B11	cultural *H. rhamnoides* ssp. *sinensis*	2n = 24	1.60–2.69 μm	1.68
B12	*E. lanceolate*	2n = 28	1.05–3.84 μm	3.66
B13	*P. americana*	2n = 24	1.26–4.36 μm	3.46
B14	*L. baviensis*	2n = 24	2.09–4.99 μm	2.39
B15	*L. elongate*	2n = 24	1.74–3.28 μm	1.89
B16	*B. diaphana*	2n = 28	2.09–2.93 μm	1.40
C1	*K. paniculate*	2n = 32	0.56–1.12 μm	2.00
C2	*J. regia* L. ‘Chuanzao1’	2n = 34	0.63–2.60 μm	4.13
C3	*J. regia* L. ‘Yanyuanzao’	2n = 34	1.01–2.55 μm	2.52
C4	*J. sigillata* Dode ‘Maerkang’	2n = 34	0.66–1.88 μm	2.85
C5	*J. sigillata* Dode ‘Muzhilinhe’	2n = 34	0.87–2.79 μm	3.21
C6	*P. macrophyllus*	2n = 36	1.92–7.01 μm	3.65
C7	*I. polycarpa*	2n = 38	0.94–2.23 μm	2.37
C8	*S. oblata*	2n = 46	1.64–2.20 μm	1.34
C9	*L. lucidum*	2n = 46	0.98–1.36 μm	1.39
C10	*L. × vicaryi*	2n = 46	1.37–3.07 μm	2.24
C11	*F. pennsylvanica*	2n = 46	0.98–2.06 μm	2.10
C12	*F. simplex*	2n = 36	1.08–2.37 μm	2.19
C13	*H. mutabilis*	2n = 90	1.22–2.90 μm	2.38
C14	*Z. armatum*	2n = 98	0.98–1.99 μm	2.03
C15	*Z*. *bungeanum*	2n = 136	0.94–2.62 μm	2.79

## Data Availability

All data and materials are included in the form of graphs in this article.
